# The Effectiveness of Nutritional Education Interventions on Dietary Intake in Young Black Males: A Near-Empty Systematic Review

**DOI:** 10.3390/nu14112264

**Published:** 2022-05-28

**Authors:** Hannah E. Jones, Crystal F. Haskell-Ramsay, Marc A. Briggs, Julie Young

**Affiliations:** Faculty of Health and Life Sciences, Northumbria University, Newcastle-Upon-Tyne NE1 8ST, UK; crystal.haskell-ramsay@northumbria.ac.uk (C.F.H.-R.); marc.a.briggs@northumbria.ac.uk (M.A.B.); julie2.young@northumbria.ac.uk (J.Y.)

**Keywords:** young people, adolescent, Black, male, African American, nutrition intervention, nutrition education, systematic review

## Abstract

The incidence of several diet and lifestyle-related diseases, previously seen only in adults, is increasing in prevalence in young people. The Black population, and particularly Black males, are at high risk of developing lifestyle-related diseases. Adolescence and young adulthood are considered a transitional period with increasing independence and responsibility, along with the development of lifelong lifestyle habits. This systematic review aimed to establish which methods and approaches to nutritional education interventions are the most effective in improving the nutritional/dietary intake in healthy young Black males. Eligibility criteria were designed using PICOS and included controlled trials of nutrition education interventions designed to improve dietary intake in healthy young Black or mixed-race males aged 14–21 years old. Medline, Cinahl and Scopus were searched in April 2021, resulting in 20,375 records being screened, and subsequently 72 full-text articles were reviewed. Risk of bias was assessed using the ROBINS-I tool. One study met the eligibility criteria. Results are presented in a narrative format as meta-analysis was not possible. This systematic review revealed a lack of evidence on the effectiveness of nutritional education interventions in this high-risk population. Limitations are noted and recommendations have been made.

## 1. Introduction

The importance of good nutrition throughout all life stages is well established and a poor nutritional intake has implications for the development of many health issues including non-communicable diseases such as cancers, cardiovascular diseases and stroke [1]. Many diet- and lifestyle-related health issues such as diabetes, hypertension, obesity and other cardiovascular disease risk factors, which were previously seen developing in middle aged people are now being seen in young people with increasing frequency [2,3].

People who are Black have a higher prevalence of some diet- and lifestyle-related conditions, especially hypertension, and Black men in particular appear to be at high risk. The Center for Disease Control (CDC) in the USA reported that in 2015–2018 there was a 58.4% prevalence of hypertension in Black men, compared with 49.8% of White men, 51.9% of Asian males, 50.4% Hispanic or Latino males and 50.3% Mexican males [4]. The data show a 9.7% prevalence rate for heart disease in the Black population, compared to 11% prevalence in the White population; however, Black people aged 18–35 years are twice as likely to die of heart disease when compared to White people of the same age [4,5].

The Health Survey for England 2004 [6], which is the most recent of the published surveys to report the specific results of the health of ethnic minorities, showed a higher prevalence of both diabetes and hypertension in the Black population, compared to the general population. The prevalence of diabetes is reported to be at least 3 times higher amongst Black adults than the general population and was more prevalent at younger ages. For hypertension, Black Caribbean males in the UK had a prevalence of 38.4% and as high as 73% for those aged 55 and older, compared with 31.7% and 58%, respectively, in the general male population. Interestingly Black African males in the UK had a hypertension prevalence of 25.1% overall, which the authors suggested was likely to be related to differences in dietary sodium intakes in different ethnic groups. In Canada the prevalence of diabetes is reported to be 2.1 times in Black adults than in White adults [7].

Whilst headway is being made in the reduction in incidence of stroke in the White population in the UK (40% reduction between 1995 and 2010), this level of reduction has not been seen in the Black population in the UK. Black people are 1.5–2.5 times more likely to have a stroke, have an increased risk of death due to stroke, and are more likely to have a stroke at a younger age, compared to their White counterparts [8,9,10]. In addition, Wang et al. (2013) note that the most significant reductions in stroke were in those people over 45 years old, but any changes in younger people were not significant. Black persons were also more likely to suffer ischaemic strokes, which tend to be associated with diabetes, hypertension and obesity. They observed that improvements in the reduction in these cardiovascular risk factors has been seen in older White adults but not in younger people or the Black population. In addition, the CDC suggests that more young Black people are experiencing health conditions previously seen in older White populations. Death rates have been declining in the Black population overall, although this has been seen primarily in the older population and not the younger Black population [5].

The reasons for the continuing health disparities between different populations are unclear, and likely multifaceted, with cultural, behavioural, physiological, socio-economic and educational factors, along with differences in access to and engagement with healthcare, all likely to play a part [8,10]. Wang et al. (2013) highlight the importance of considering the differences in different populations when designing health education and prevention programs. Nutrition, or dietary intake, is a key modifiable behavioural risk factor in the development of cardiovascular diseases. The King’s Fund report [10] indicated that whilst 55–56% of the White population consumed the recommended five daily servings of fruits or vegetables, only 44% of the Black population were achieving this. The Black population are more likely than the White population to be sensitive to deficiencies in potassium intake which may exacerbate the effect of dietary sodium on blood pressure [11]. Interventions such as the Dietary Approaches to Stop Hypertension (DASH) diet have shown to be particularly effective in reducing blood pressure in the adult Black population [12].

Children over the age of 14 may be less dependent than younger children on their parents for their dietary intakes [13]. The period of adolescence and young adulthood is often considered a transitional period where many changes, including mental, social and physical changes in the individuals’ life, are happening. These transitions include changes in education or leaving school, entering employment or becoming unemployed, or becoming a parent [14]. Adolescence may be a prime time for nutrition and lifestyle education and personal skill development [15]. Nutrition and lifestyle education is an important primary prevention strategy to reduce the risk of individuals developing diet- and lifestyle-related conditions in the future. Interventions focusing on young people can be more effective at prevention as the disease(s) have not yet presented [14]. Whilst preventative education is often population based, the aim is to change the behaviour of individuals. The importance of targeted, tailored, culturally acceptable interventions for different populations to increase effectiveness is well documented [9,14,16].

### Objectives

This systematic review was designed to establish the effectiveness of different nutritional education interventions on nutritional intake in healthy young Black males aged between 14 and 21 years old and further establish which methods and approaches are the most effective in improving the nutritional/dietary intake in this population.

## 2. Methods

The protocol for this systematic review was registered prior to commencement with the PROSPERO International prospective register of systematic reviews (Registration number CRD42021247726). It is reported using the Preferred Reporting Items for Systematic Reviews and Meta-Analyses (PRISMA) 2020 guidelines [17]. Eligibility criteria were designed using PICOS for Inclusion and Exclusion Criteria [18]. Studies were included or excluded if they met any of the following eligibility criteria.

### 2.1. Population

Inclusion: Black or mixed-race males 14–21 years old who do not have any acute or chronic illness.

Exclusion: Non-Black; Black or mixed-race people under 14 years old or over 21 years old; Black or mixed-race males aged 14–21 years old whose data cannot be clearly separated in the study. People with an identified acute or chronic illness such as cardiovascular diseases, cancers, gastrointestinal conditions, eating disorders or intellectual disabilities. Research in low-income countries was excluded.

### 2.2. Intervention

Inclusion: Nutrition education intervention given to improve dietary intake. Multiple types of nutritional education interventions such as 1:1, face to face, groups, telehealth, paper-based and app-based were included.

Exclusion: Nutritional education or other nutritional intervention given to treat specific medical issues such as cardiovascular diseases, cancers, gastrointestinal conditions, eating disorders, endocrine disorders or intellectual disabilities. Use of nutritional supplements or infusions. Studies where the nutritional intervention/education methodology is not well described. Studies that focus only on one or more nutrients, rather than whole foods or macronutrients.

### 2.3. Comparisons/Control

Inclusion: Control groups should have no intervention or a clearly defined ‘standard treatment’.

Exclusion: No control group, poorly defined intervention or treatment for control group.

### 2.4. Outcomes

Inclusion: Changes in the nutritional content of the diet consumed (whole foods or macronutrients), nutritional knowledge and mental wellbeing.

Exclusion: Non-nutritional/dietary outcomes as the primary outcome.

### 2.5. Study Design

Inclusion: RCTs, randomised cluster trials, non-randomised controlled studies and cohort observational studies.

Exclusion: Cross-over trials, before-and-after studies, interrupted time series, case–control studies, case series and other studies.

The electronic databases CINAHL, SCOPUS and Medline including PubMed (via ProQuest) were searched between 21 April and 26 April 2021 to produce a comprehensive search from inception. Filters and limits included (where possible) English language, human(s), published in academic journals and peer-reviewed studies.

### 2.6. Keywords/Search Terms

The following keywords and search terms were used (See Table 1). These terms include relevant synonyms for the original search terms to ensure all potentially relevant articles were identified.

### 2.7. Search Terms

(“Young people” OR “Young persons” OR Adolescent * OR Teenage * OR Youth * OR Juvenile *) AND (Black * OR African American * OR Afr *-Caribbean * OR “BAME”) AND (Male * OR Men OR Boy *) AND (Nutrition * OR Diet * OR Food * OR “Medical Nutrition *”) AND (Intervention * OR Education * OR Therap *) NOT (Supplement * OR Infusion *). Note: SCOPUS requires AND NOT, rather than NOT.

A total of 81,066 records were identified from the databases; Medline (n = 367), CINAHL (n = 257) and SCOPUS (n = 80,442). In order to download the SCOPUS results as a CVS file for input into Endnote for processing it was required that we further filter the results to a maximum of 20,000 results. Firstly, keyword filters were added to limit results to controlled study, major clinical study, cohort analysis, RCT, prospective study, prospective studies, cohort studies and controlled clinical trial. Secondly, keyword filters to exclude the terms middle aged, preschool child, child preschool, infant, aged 80 and over, very elderly, newborn, infant newborn, human tissue, non-human, pregnancy and human cell. Medline and CINAHL results were downloaded in full.

The resulting 20,536 studies identified using the above search strategies were imported into EndNote X9.3.3 (EndNote software is managed by Clarivate, Philadelphia, PA, United States) by the primary researcher (HJ) to catalogue and organise the studies. Using the Endnote X9 software 36 duplicates were identified. All 20,536 studies were then uploaded to the Rayyan QCRI web application for systematic reviews [19] where they were screened again for duplicates and 155 duplicates were identified. These were manually cross-checked with the Endnote duplicates for accuracy and consequently a total of 161 duplicate studies were removed. The remaining 20,375 studies were screened for eligibility using the pre-defined inclusion and exclusion criteria. Titles and abstracts were individually screened by the primary researcher for relevance and cases of any uncertainty, the secondary researcher (J.Y.) was consulted. The secondary researcher individually screened the titles and abstracts of those studies where there was uncertainty and then the researchers verbally discussed and resolved any conflicts. This primary screening process resulted in 20,303 exclusions and 72 studies which were further screened for eligibility using the full text articles.

Articles were included for full text screening if the abstracts did not clearly identify the characteristics of the study group including race, age and health status, interventions and outcome measures. Full text articles were retrieved for the remaining 72 studies and reviewed again by the primary researcher for eligibility with the aim of creating the final list of studies to be included. In the case of any uncertainty the secondary researcher was consulted and the papers discussed.

**Figure 1:**
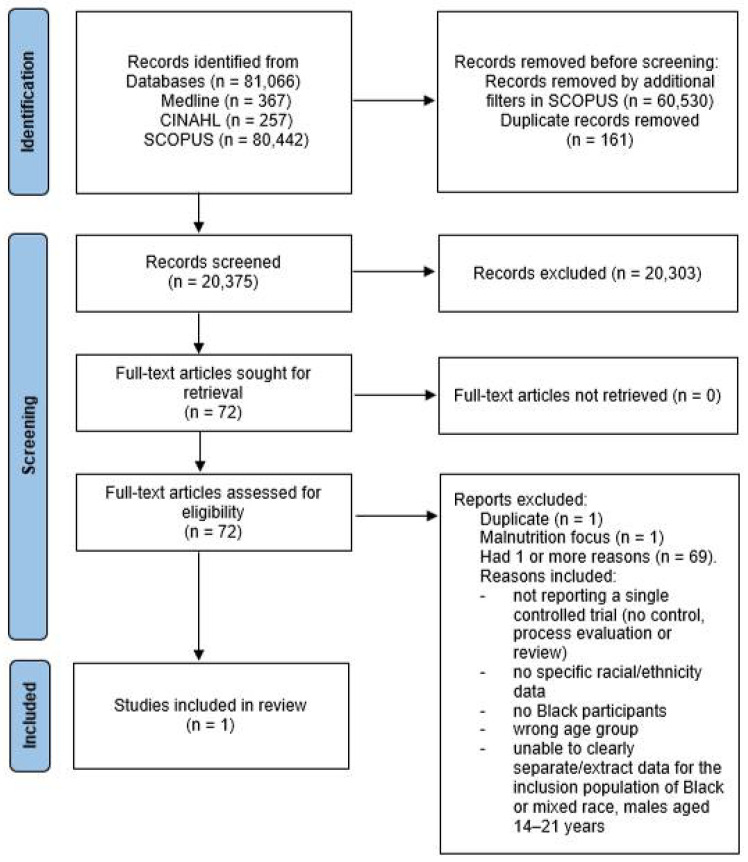
PRISMA Flow Diagram.

Only 1 paper met the inclusion criteria and 71 papers were excluded. One paper was excluded as it was identified as an additional duplicate and one paper was excluded as it focused on malnutrition in low-income communities within South Africa and was therefore not relevant to this review. The remaining papers were excluded for one or more of the following reasons: not reporting a single controlled trial (no control, process evaluation or review); no specific racial/ethnicity data; no Black participants; the study was focused on the wrong age group; and/or the data were presented in a form where it was not possible to clearly separate or extract the specific, relevant data for the inclusion population of Black or mixed race, males aged between 14 and 21 years old. (See Figure 1).

As meta-analysis is not possible, results are presented in a narrative format.

Risk of bias in the single eligible study was assessed by the primary researcher using the Cochrane ROBINS-I tool (Risk of Bias in Non-randomized Studies—of Interventions) and the associated detailed guidance document [20,21]. This risk of bias tool is designed for cohort-type studies. This is a deviation from the published PROSPERO protocol in which it was intended to use the revised Cochrane risk of bias tool for randomized trials (RoB 2) [22]; however, the ROBINS-I tool was deemed more appropriate for the quasi-experimental, cohort design used in the study.

## 3. Results

Despite an extensive and systematic review of the published literature, only one paper met the eligibility criteria and was included in this review. This paper [23] describes an investigation of a health promotion intervention that was targeted at African American adolescents at a mostly Black school in Florida, USA. Participants were recruited from two ‘required courses’ and hence were considered representative of the school population. Prerequisites for inclusion were being 14–17 years old, African American (Black) and the ability to read and write in English. Consent and assent were required from parents and children, respectively. Relevant aims of the intervention were to increase intakes of fruits and vegetables, and increase knowledge of health promotion. Other aims were to maintain normal blood pressure and increase exercise levels.

The assessments were developed by the principal investigator and were completed at weeks 1 and 9, and included a health knowledge test and a 2-day dietary recall/questionnaire, along with a blood pressure measurement. Participants in the intervention group received, within their usual two 90 min classes per week, one exercise-focussed class and one class focussed on cognitive behavioural concepts related to the study aims. The control group had no change to their usual classes, other than the pre- and post-assessments.

### Risk of Bias

**Figure 2:**
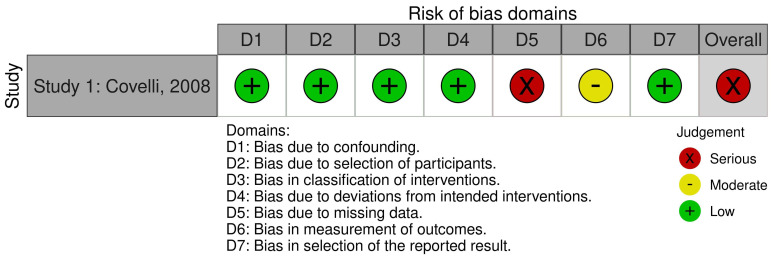
Risk of Bias ‘Traffic light’ plot.

A risk of bias assessment, using the ROBINS-I tool, was conducted on the single paper and the robvis (visualization tool for risk of bias assessments in a systematic review) [24] was used to create a ‘traffic light’ plot. (See Figure 2). Seven domains of bias were assessed: bias due to confounding; bias in selection of participants into the study; bias in classification of interventions; bias due to deviations from intended interventions; bias due to missing data; bias in measurement of outcomes; bias in selection of the reported result; plus the overall judgement of bias. Risk of bias in the first four and final domains was deemed to be low. Bias due to missing data was deemed serious due to high levels of attrition. A total of 16% of intervention participants and 32% control participants were lost (23% overall); however, baseline data only provided for those who completed the study and there was no assessment of the effects of attrition on the results. Since the study was small, the effects of this loss of data could be significant. Bias in measurement of outcomes was deemed moderate due to lack of blinding and the principal investigator’s involvement in weekly assessments of the intervention through participant journals and in making recommendations. Overall, this paper falls in the serious, but not critical, risk of bias category since one domain was judged to be at serious risk of bias. This is not unexpected for a quasi-experimental study [21].

Limited data which met the inclusion criteria were available; however, they did report on changes in the number of fruits and vegetables consumed each day. The paper also reported on changes in health knowledge scores which included, amongst other things, the effects of nutrition lifestyle behaviours on health.

Health knowledge scores were based on a 20-item true or false test which was created by the principal investigator for that study. The questions included multiple topics and it was not stated how many of the questions related to the effects of nutrition lifestyle behaviours on health. We are therefore unable to confidently assess any changes in nutritional knowledge, as per our review protocol.

Changes in fruit and vegetable intakes are reported as raw data, although the unit of measurement is not specified. For the purpose of this review, we have assumed standard portion sizes. They report males in in intervention group consuming 2.8 units per day at baseline, increasing to 4.9 units per day on follow-up. Males in the control group consumed 2.9 units per day at baseline, and 2.7 units per day on follow-up. The data analysis with *t* tests presented did not separately identify males and female participants and therefore cannot be reported. It was also stated that intakes of high sodium foods were also assessed via the 2-day dietary recall/questionnaires; however, no results of this nature were reported.

## 4. Discussion

Despite an extensive and systematic review of the published literature, only one paper met the eligibility criteria; thus, we have presented a near-empty systematic review. This systematic review revealed a lack of literature on effectiveness of different nutritional education interventions on nutritional intake in healthy young Black males. This was a surprising discovery given the widespread acknowledgement that the Black population, and particularly Black males, are at high risk for developing diet- and lifestyle-related non-communicable diseases. Research highlighting these health risks and calls for targeted educational interventions for the Black population have been well documented over many decades [25,26], with research documenting the elevated blood pressure of Black men in comparison with their White counterparts as long ago as 1932 [27]. The current review also highlights the challenges in extracting relevant information and data when sub-analysis of participants is not conducted. Several themes emerged from this systematic review process.

There were several reasons why studies were not deemed eligible for inclusion. A number of papers did not have any control group [28,29,30]. Several studies did not report on race, but rather different ethnicities or language spoken at home [31,32,33,34]. Several papers did report demographics including gender, ages and Black or African American race; however, whilst ensuring intervention and control groups were similar and representative of their population, the results were not presented in such a way that the results for young Black males could be isolated or distinguished from the overall intervention or control groups [15,28,35,36,37]. If such sub-group analysis was completed, it was not presented in the paper. Several papers simply involved low numbers of Black participants; for example, studies with 4% [38], 9% [39], 10% [40], 12% [41], and less than 15% [29] of participants who completed the study were Black. There were no Black participants in a number of studies [42,43,44]. Some papers included high proportions of, or all Black participants; however, the results were presented only for intervention and control, and there was no sub-analysis of gender and/or age specific results [45,46].

One can speculate about the reasons for the challenges in identifying data that are relevant or specific to the young, Black male population of this systematic review. One widely recognised challenge is the understandable reluctance of the Black population to participate in research due to historic violations such as that which occurred in the Tuskegee Syphilis Study. Additional reasons may include lack of awareness of the potential vulnerability of this population developing lifestyle-related diseases, unintentional racial discrimination, insufficient participant numbers to power sub-analysis or protect the anonymity of participants, time constraints in either recruitment or data analysis, and word count limitations for publications. This would be an interesting retrospective investigation in itself. Recommendations for research made by NICE (2007) included researchers and commissioners identifying and accounting for differences in the effectiveness of interventions amongst different social and ethnic groups, ages and genders.

It was not possible in this review to definitively establish which methods and approaches are the most effective in improving the dietary intake in the healthy young, Black male population. More generally speaking, interventions which included policy changes at a school, district or national level seemed to be the most effective [38,47]. This strategy ensures that the majority of school-aged children would receive nutritional education, which may have a positive impact upon their current and future health. Other important considerations for improving the effectiveness of nutritional education interventions appear to be tailoring, and particularly cultural and individual, dynamic, ongoing tailoring [46,47,48,49] and goal setting with individual participants, in particular one-to-one consults with a specially trained counsellor [28]. Even within individual studies there were clear differences in the effectiveness between male and female participants [32,50]. There was no benefit of family-based interventions [51,52] and parent participation in school-based interventions for adolescents can be poor [36]. Complex, multi-level interventions present challenges in determining which factors were influential in any outcome effects [46] and there is no clear consensus of whether interventions which target single or multiple behaviours are more effective. There did not appear to be any clear consensus of an optimal duration or dosage of interventions. The majority of interventions incorporated one or more behaviour change theories, but again, there is no clear consensus in which method is more efficacious. Producing improvements in participants’ knowledge and intake of fruits and reductions in nutritionally poor snack foods appears to be less challenging than producing improvements in participants’ knowledge and intake of vegetables [32]. Improvements in knowledge occur more readily than changes in behaviour [47] but it is widely accepted that knowledge is a precursor for behaviour change. In addition, many of the positive, statistically significant effects on dietary intake have been relatively small and one has to question whether there is a clinical significance of some of these interventions.

Interventions which have used CD-ROM and even text messaging may already be less relevant due to the fast-moving pace of technological advances and consequently the communication preferences of adolescents and young adults. With that in mind, it is important to continually review the effectiveness of tailored nutritional education interventions and request feedback from participants to ensure they remain relevant and able to produce optimal results.

A large proportion of the papers which were excluded in the primary screening process of this systematic review were deemed ineligible because they aimed to treat specific medical issues. We believe that it is fundamentally important to provide appropriate, effective nutritional education to this high-risk population before they develop a diet- and lifestyle-related disease. This is important to benefit not only the health of these individuals, their families and communities, but also reduce the economic cost burden for those people, the healthcare system, welfare system and wider economy [53].

Limitations of this review include the inclusion only of English language, peer-reviewed papers published in academic journals, and we did not seek papers in other languages, nor grey literature. Another limitation is that this systematic review focused on a specific age group (adolescents and young adults) and male gender of the Black population; however, given the health risks of this population, the authors believe it is justified. In addition, given the eligibility criteria included other commonly used study types and did not limit to randomized controlled trials, and the large number of results from the initial searches, we feel that the criteria were appropriate. It is probable that research needs to be conducted in other age brackets and with females to ensure nutritional, and other health education can be effectively targeted and tailored to meet the needs and minimise the health risks of specific populations. People who are Black have a higher prevalence of some diet- and lifestyle-related conditions, especially hypertension, and Black men, in particular appear to be at high risk [4]. The CDC data show that even in conditions where the prevalence is lower in the Black population compared to the White population, Black people are more likely to die of that condition [4,5].

## 5. Conclusions

This systematic review revealed a lack of literature on effectiveness of different nutritional education interventions on nutritional intake in healthy young Black males. This does not mean that current or previously investigated interventions are, or are not, effective for this population, rather that the literature has not been analysed in such a way that we have any evidence that they are effective in this population.

This review indicates that research is needed to establish effective prevention and treatment interventions for young, Black males to ensure that their health risks are reduced and healthy life expectancy improved. In addition, secondary analysis of previous research data should be conducted to identify and compare the effectiveness of interventions across different combinations of sub-sets of race, ethnicity and gender.

## Figures and Tables

**Table 1:** Keywords.

Population	Interventions	NOT
Young people Young persons Adolescent * Teenage * Youth * Juvenile * Black * African American * Afr *-Caribbean * BAME Male Men Boy *	Nutrition * Diet Food * Intervention * Education * Medical Nutrition * Therap *	Supplement * Infusion *

* was used for truncation purposes.
